# Advancements in Targeted Therapies for Colorectal Cancer: Overcoming Challenges and Exploring Future Directions

**DOI:** 10.3390/cancers17172810

**Published:** 2025-08-28

**Authors:** Said A. Khelwatty, Soozana Puvanenthiran, Alan M. Seddon, Izhar Bagwan, Sharadah Essapen, Helmout Modjtahedi

**Affiliations:** 1 School of Life Science, Pharmacy and Chemistry, Kingston University London, London KT1 2EE, UK; alan.seddon@kingston.ac.uk (A.M.S.); izhar.bagwan@nhs.net (I.B.); h.modjtahedi@kingston.ac.uk (H.M.); 2 Faculty of health and Life Sciences, Oxford Brookes University, Oxford OX3 0BP, UK; p0093087@brookes.ac.uk; 3 Berkshire Surrey Pathology Services, Royal Surrey Hospital, Guildford GU2 7XX, UK; 4 St Luke’s Cancer Centre, Royal Surrey County Hospital, Guildford GU2 7XX, UK; s.essapen@nhs.net

**Keywords:** colorectal cancer, targeted therapy, HER family

## Abstract

Colorectal cancer (CRC) remains a significant global health burden. While early-stage CRC has a high survival rate, most patients are diagnosed with advanced disease, necessitating more effective and less toxic therapeutic targets. This review examines recent advancements, challenges, and future directions in targeted therapies for CRC, focusing on HER inhibitors. We assess the efficacy of monoclonal antibodies (mAbs) and tyrosine kinase inhibitors (TKIs) and explore strategies to overcome resistance mechanisms. Targeted therapies like cetuximab and panitumumab have improved outcomes for CRC patients with wild-type KRAS. However, resistance mechanisms and intra- and inter-tumour heterogeneity limit their effectiveness. Recent advancements include the development of dual TKIs, antibody/drug conjugates (ADCs), bispecific antibodies, and CAR-T cells against HER family members and other targets that are showing promise in preclinical and clinical trials. Targeted therapies have transformed CRC treatment, but more research is needed to overcome some of the current challenges, such as late diagnosis and the heterogenous nature of CRC, as well as the discovery of more reliable biomarkers for response to the therapy and patient selection. Future research should focus on identifying novel biomarkers of diagnostic, prognostic, and predictive value, developing next-generation inhibitors, drug repurposing, and combining small-molecule targeted therapies with immunotherapy. Such advances could ultimately help increase both the treatment options and outcomes for patients with CRC.

## Simple Summary

Despite the approval of several targeted therapeutic agents for the treatment of cancer patients, colorectal cancer is a major health problem. It is essential to discover additional and colorectal cancer-specific therapeutic targets. Moreover, for the past few decades, we and others have been working on the development of more effective and less toxic therapeutic interventions for patients with colorectal cancer. One promising area is targeting HER family proteins, which help cancer cells grow. This review article highlights advances in the development of various drugs that block HER proteins and other proteins that are at different stages of clinical development, and the therapeutic potential of such drugs when used in combination to overcome current challenges, such as poor response or no response. The goal is to find treatments that are more effective and have fewer side effects, ultimately improving the quality of life of patients receiving treatment for colorectal cancer.

## 1. Introduction

With an estimated 1.9 million new CRC cases worldwide and 935,000 deaths in 2020, CRC remains a major health burden globally. CRC is the third most diagnosed cancer but the second leading cause of cancer deaths, which represents around 1 in 10 cancer cases and deaths [1]. However, there are variations in both the incidence and mortality rates of CRC worldwide. CRC is now considered a marker of socioeconomic development, and as such, incidence rates are highest in European regions, North America, Australia, and New Zealand, while incidence tends to be lower in Africa and South Central Asia [2,3].

While early-stage colorectal cancer carries an excellent prognosis, with five-year survival approaching 90%, most patients present with either locally advanced or metastatic disease. In these settings, outcomes remain poor—five-year survival falls to around 68% for locally advanced cases and just 10% for metastatic disease [4,5]. This highlights the vital need to identify precise biological and molecular targets that can guide the development of more effective and less toxic therapies.

Generally, metastatic colorectal cancer (mCRC) therapy has been based on fluoropyrimidine (e.g., 5-fluorouracil) chemotherapy and drugs such as irinotecan and oxaliplatin introduced later to this already established chemotherapeutic regimen [6]. While these first- and second-line treatments have led to better prognosis in mCRC patients, their most prominent drawback is the non-specificity and toxicity of the treatment, which ultimately leads to various adverse effects such as haematological disorders and increased risk of infections [7,8,9,10].

To overcome these challenges, it has been recognised that a better understanding of CRC biology, together with the discovery of more specific therapeutic targets, could lead to the development of more specific and less toxic targeted therapy for patients with mCRC. Of various biomarkers that can be targeted, epidermal growth factor receptor (EGFR) was the first therapeutic target identified in patients with CRC. Today, the aberrant expression of growth factor receptors on the cell surface of various human cancers is regarded as a major target for therapy with various forms of monoclonal antibody (mAb)-based products and small-molecule TKIs. We previously published a comprehensive review on the expression patterns, prognostic significance, and predictive value of HER family members for response to therapy with HER inhibitors in CRC [11].

This review is intended to provide an update to some advancements, in particular, the recent FDA approvals of new drugs, challenges and future directions in the development of targeted therapies of CRC using drugs targeting HER family members and other biomarkers, and the importance of biomarkers of predictive value in the selection of a more specific population of CRC patients who benefit from such therapeutic interventions.

## 2. Advancements in Targeting CRC

### 2.1. Discovery of the Type I Growth Factor Receptor and Its Ligands

One of the major advancements in the targeted therapy of cancers, including CRC, was the discovery of the type I growth factor receptor family of tyrosine kinases, which consists of EGFR (HER1, ErbB-1), HER2 (neu, ErbB-2), HER3 (ErbB-3), and HER4 (ErbB-4), of which, EGFR and HER2 are the most extensively studied receptors [12,13,14].

**Figure 1:**
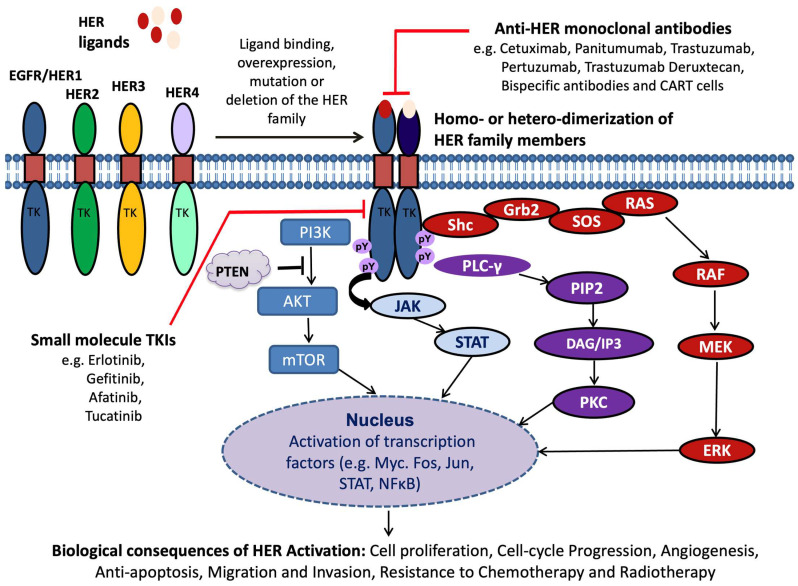
Activation and signalling of HER family members and their targeting using various types of mAb-based drugs and small-molecule TKIs. The increased activation of the HER signalling pathways is mediated via the increased expression of ligand binding, overexpression, mutation, and/or deletion of the HER receptors, leading to receptor dimerization (homo- or hetero-), activating intrinsic tyrosine kinase activity within the receptor’s C-terminal domain. This leads to tyrosine phosphorylation (pY) within the tyrosine kinase domain (TK) and subsequent activation of downstream signalling cascades that regulate a range of cellular processes, including cell proliferation and survival, differentiation, apoptosis inhibition, angiogenesis, maturation, cell adhesion, and invasion. These can be targeted using various forms of inhibitors targeting the external or internal domains of these receptors. Adapted from reference [17].

These receptors are activated following the binding of several ligands such as epidermal growth factor (EGF), transforming growth factor alpha (TGFα), amphiregulin (AR), heparin-binding betacellulin (BTC), and epiregulin (EPI) to its extracellular domain [15,16]. The formation of dimers (homo- or hetero-) between different HER family members is brought about by the binding of cognate ligands to the external domain of these receptors. Consequently, the autophosphorylation of numerous tyrosine residues leads to the activation of downstream signalling molecules, such as the RAS, RAF, and mitogen-activated protein kinase (MAPK) cell proliferation pathways and the phosphatidylinositol 3 kinase (PI-3K)/Akt cell survival pathway, as shown in Figure 1.

In addition, HER family members, and in particular EGFR, activates other important downstream signalling pathways such as janus kinase and signal transducer and activator of transcription (JAK/STAT) and phospholipase C (PLC-γ)/Ca^2+^/calmodulin-dependant kinases, consequently leading to increased cell proliferation, reduced apoptosis, increased angiogenesis, migration, invasion, and resistance to chemotherapy and radiotherapy, which are the hallmarks of human cancer [13,18,19,20,21,22,23,24].

As a result, the first generation of HER inhibitors, namely anti-HER2 mAb trastuzumab (1988), anti-EGFR mAbs cetuximab (2004) and panitumumab (2006), and EGFR TKIs gefitinib (2003) and erlotinib (2004), were developed and approved for treatment of patients with breast, colorectal, and non-small cell lung cancer, respectively [25,26,27,28]. These and the newer generation of HER inhibitors will be discussed below.

### 2.2. Development of Anti-HER Inhibitors

In the past few decades, there have been advances in our understanding of the complex biology of human cancers and the immune system, and technological advances, which have translated into the approval of novel therapeutics. Indeed, we have witnessed rapid advancements in the development of second, third, and fourth generation HER inhibitors, not only in the form of mAbs and TKIs but also other types of HER inhibitors, including dual EGFR/HER2 TKIs, pan-HER TKIs, ADCs, and bispecific antibodies, as well as immunotherapies such as CAR-T cell targeting HER family members.

#### 2.2.1. EGFR Targeted Therapy

Over the past two decades, the landscape of targeted therapy for human cancers has evolved significantly, largely due to advancements in tumour molecular profiling technologies that have transformed the treatment of mCRC. In the realm of mCRC, the two most frequently utilised anti-EGFR targeted therapies are the FDA-approved mAbs cetuximab and panitumumab, which are often administered in conjunction with chemotherapy [27,29,30,31,32,33,34,35,36,37,38]. Additionally, small-molecule TKIs have been thoroughly investigated in CRC. Beyond this, they have also secured approval for the treatment of non-small cell lung cancer (with gefitinib and erlotinib) and pancreatic cancer (with erlotinib) [39,40,41]. A variety of other EGFR inhibitors, including dual EGFR/HER2 TKIs and pan-HER TKIs, are currently undergoing various stages of preclinical and clinical trials, some of which will be examined in further detail herein (Table 1).

##### Cetuximab (Erbitux^®^)

Cetuximab is a chimeric IgG1 mAb that selectively binds to the external domain of the EGFR, inhibiting ligand binding and subsequent receptor activation and tyrosine kinase activity [29,42]. As an IgG1 antibody, cetuximab also induces in vivo anti-tumour effects through antibody-dependent cellular cytotoxicity (ADCC) and complement-dependent cytotoxicity (CDC).

The BOND trial by Cunningham et al. [27] demonstrated the efficacy of cetuximab in chemo-refractory mCRC. In this study, 329 patients with disease progression during or within three months of irinotecan treatment were randomised into two groups: 218 received cetuximab plus irinotecan, while 111 received cetuximab alone. The overall response rate was 22.9% for the combination therapy versus 10.8% for monotherapy (*p* = 0.007). Moreover, median time to progression was significantly longer for the combination group (4.1 months) compared to cetuximab alone (1.5 months). However, there was no significant difference in median overall survival rates (8.6 months vs. 6.9 months, *p* = 0.48). Notably, four patients experienced severe anaphylactic reactions to cetuximab, leading to treatment cessation. Following the BOND study, cetuximab gained FDA approval for use in irinotecan-refractory mCRC [29].

**Table 1:** Summary table of EGFR family inhibitors currently under preclinical and clinical investigation in CRC.

Agents	Properties	Status	Reference/Clinical Trial
**Antibodies**			
Cetuximab	Chimeric anti-EGFR (IgG1)	Approved for CRC, head and neck cancer	[29]
Panitumumab	Fully human anti-EGFR (IgG2)	Approved in 2006 for CRC	[43]
Nimotuzumab/ h-R3	Humanised anti-EGFR (IgG1)	Phase II in CRC	NCT00972465
Necitumumab/ IMC-11F8	Fully human anti-EGFR (IgG1)	Phase II in CRC	NCT00835185
ICR62	Rat anti- EGFR (IgG2b)	Preclinical/phase I in solid tumours	[44,45,46]
Trastuzumab/ Herceptin	Humanised anti-HER2 (IgG1)	Approved for breast cancer Phase II in mCRC	ref. [47], NCT04744831, NCT03457896, NCT05193292
Pertuzumab/ 2C4	Humanised anti-EGFR and HER2	Phase I/II in CRC	NCT00551421
Seribantumab/ MM-121	Fully human anti-HER3	Phase I in advanced/solid tumours	NCT01451632, NCT01451632, NCT01447225, NCT04383210
MM-111	Bispecific anti-HER2 and HER3	Phase I in solid tumours	NCT00911898
MCLA-158	Bispecific anti-EGFR and LGR5	Phase I in advanced tumours and CRC	NCT03526835
Sym-004	Anti-EGFR mAb Mixture	Phase II in mCRC/solid tumours	NCT02083653, NCT01117428
MEHD7945A	Humanised anti-HER3 (IgG1)	Phase I/II in metastatic or advanced cancers and CRC	NCT01986166, NCT01652482
MM-151	Anti-EGFR mAb Mixture	Phase I in solid tumours	NCT01520389
**Small Molecules**			
Gefitinib	Reversible anti-EGFR	Phase II/III in CRC Approved for NSCL cancer	ref. [25], CT00234429
Erlotinib	Reversible anti-EGFR	Phase II/III in CRC Approved for NSCL cancer and pancreatic cancer	ref. [28], NCT00598156, NCT01229813
Afatinib/ Gilotrif	Irreversible ErbB family blocker	Phase II in CRC; Approved for NSCLC in 2013	ref. [48], NCT01152437, NCT02020577
Canertinib	Pan HER inhibitor	Preclinical in CRC	[49]
HM781-36B	Pan HER inhibitor	Phase I solid tumours	NCT01455584, NCT01455571
Lapatinib	Reversible anti-EGFR and anti-HER2	Approved for Breast Cancer Phase II in CRC	[50]
BMS-599626	Pan HER inhibitor	Phase I in solid tumours	ref. [51], NCT00095537
EKB-569	Irreversible anti-EGFR	Phase I/II in CRC	NCT00072748
Neratinib/ Nerlynx	Pan HER inhibitor	Phase I in solid tumours, approved in HER2-positive metastatic breast cancer in 2020	NCT03919292
Tucatinib/ Tuksya	Anti-HER2 inhibitor	Approved in CRC	[52]
Vandetanib	Pan VEGFR/EGFR/RET inhibitor	Phase I/II in CRC	NCT00532909, NCT00500292, NCT00507091

The effectiveness of cetuximab is confined to patients with wild-type KRAS tumours. Approximately 40% of CRC cases involve KRAS mutations, which correlate with a poor prognosis [53]. In cases of KRAS wild-type metastatic liver disease secondary to CRC, adding cetuximab to standard chemotherapy has led to significantly increased response rates and liver resection rates. The CELIM study [54], a randomised phase II trial, showed a response rate of 70% for KRAS wild-type patients receiving cetuximab compared to 41% for those with mutant KRAS (*p* = 0.008). This resulted in an increase in resection rates from 32% to 60% post-chemotherapy (*p* < 0.0001).

The CRYSTAL trial [55] was the first randomised study to show a significant benefit from adding cetuximab to folinic acid, fluorouracil, and irinotecan (FOLFIRI) chemotherapy in previously untreated patients with mCRC. KRAS testing was not routinely performed at the start of this study, but the subsequent follow-up study with RAS testing highlighted the significantly increased median progression-free survival in those patients with RAS wild-type cancers treated with FOLFIRI and cetuximab compared to patients treated with FOLFIRI alone (11.4 vs. 8.4 months). Similarly, in the Van Cutsem follow-up review in 2014, there was a significantly increased overall survival (OS) in the cetuximab group vs. the FOLFIRI alone patients (28.4 vs. 20.2 months). In those patients with RAS mutant cancers, there was no increased PFS or OS in patients treated with FOLFIRI and cetuximab vs. those patients receiving FOLFIRI alone (7.4 vs. 7.5 months and 18.4 vs. 17.7 months, respectively) [56]. The CRYSTAL study was pivotal in showing the survival advantage of adding an anti-EGFR mAb to standard FOLFIRI chemotherapy, but only in those patients with molecularly selected cancers. It was one of the first studies to demonstrate the need for more personalised therapy, and led to the mandatory testing for RAS mutations to decide on the addition of the targeted mAb, cetuximab, to conventional chemotherapy to increase the patient’s survival and response rates.

The EPIC trial [57] assessed cetuximab in previously untreated patients and demonstrated significant improvements in progression-free survival and response rates when cetuximab was combined with irinotecan compared to irinotecan alone (3.9 months vs. 2.56 months; response rates of 16% vs. 4%).

Heinemann and colleagues [58] investigated the comparative effectiveness of cetuximab and the anti-vascular epidermal growth factor-A (VEGF-A) antibody bevacizumab in combination with the chemotherapy regimen (FOLFIRI) in the FIRE-3 study, which included 592 patients with KRAS exon 2 wild-type tumours. The study found median overall survival of 28.7 months vs. 25.0 months for the cetuximab and bevacizumab groups, respectively, suggesting that FOLFIRI plus cetuximab could be the preferred first-line regimen for patients with KRAS exon 2 wild-type mCRC. In addition, in a follow-up study analysing the final survival and per-protocol analysis of FIRE-3, while cetuximab plus FOLFIRI was associated with longer OS (31 vs. 26 months) compared to bevacizumab, the advantage of cetuximab over bevacizumab occurred only in patients with left-sided primary tumours [59]. However, further analysis has shown that OS was generally shorter in older patients (≥65 years) with right-sided tumours in comparison to younger patients (<65 years) with left-sided tumours treated with cetuximab plus FOLFIRI [60].

This difference in left-sided versus right-sided metastatic colon cancer in terms of biology, prognosis, and response to treatment is well described by Venook and colleagues [61]. From this and other studies, we know that left-sided mCRCs tend to have a better prognosis, are EGFR-driven, and therefore, potentially benefit significantly from the addition of an anti-EGFR mAb to standard chemotherapy. This mAb can lead to a significantly improved PFS and OS in those patients with RAS wild-type cancers.

In addition, another randomised phase 2 DEEPER trial, which enrolled 359 patients with RAS wt mCRC, tested the superiority of modified (m)-FOLFIRI plus weekly cetuximab over bevacizumab. The post hoc exploratory analysis showed that cetuximab produced greater PFS and OS in patients with left-sided and RAS/BRAF wt tumours [62]. More recently, another phase 3 trial, using cetuximab β, which is a biosimilar antibody of cetuximab, found statistically significant improvements in median PFS, OS, and ORR in groups of Chinese patients treated with cetuximab β plus FOLFIRI compared to FOLFIRI alone while maintaining a manageable safety profile [63]. It is important that future treatment regimens should integrate molecular insights to better guide personalised treatments, especially anti-EGFR therapies such as cetuximab in patients with mCRC [64,65].

##### Panitumumab (Vectibix^®^)

Panitumumab is distinguished from cetuximab as it is a fully human IgG2 mAb targeting the EGFR [66,67]. It received approval from the FDA for the treatment of mCRC that had shown resistance to 5-fluorouracil, oxaliplatin, and irinotecan-based regimens, following a comprehensive randomised open-label study led by Van Cutsem et al. This pivotal phase III trial included 463 patients, with some receiving panitumumab alongside best supportive care (BSC) and others receiving BSC alone. The study revealed a notable 56% reduction in the relative progression rate for those treated with panitumumab, while overall survival rates remained statistically comparable between the two groups [43]. Moreover, patients in the panitumumab group experienced a longer mean progression-free survival of 13.8 weeks, compared to 8.5 weeks for those on BSC alone.

Hecht and colleagues [68] conducted a non-randomised phase II multicentre study involving 148 patients with mCRC resistant to fluoropyrimidines, oxaliplatin, or irinotecan. The participants were categorised based on their EGFR expression levels. Findings indicated a modest 9% overall response rate, with 29% of patients achieving stable disease, a median overall survival of 9 months, and a progression-free survival duration of 14 weeks.

The PRIME study, a landmark phase III trial spearheaded by Siena et al. [69], was the first to assess the efficacy of combining panitumumab with 5-fluorouracil, leucovorin, and oxaliplatin (FOLFOX4) as a front-line treatment in mCRC. This study enrolled 1150 treatment-naive patients, who were randomised to receive either the combination therapy or FOLFOX4 alone, with progression-free survival as the main endpoint. This study again demonstrated a significantly increased median PFS in mCRC patients with RAS wt cancers who received FOLFOX and panitumumab compared to those who received FOLFOX alone—9.6 vs. 8.0 months (*p* = 0.02). In this study, there was a trend towards an improvement in survival in the panitumumab group, but this did not reach statistical significance—23.9 months vs. 19.7 months (*p* = 0.07). Initial safety analyses revealed neutropenia (28%) and diarrhoea (11%) to be the predominant adverse effects, with 10% of participants reporting grade 3 skin reactions.

To date, there are no back-to-back studies looking at the relative merits of FOLFIRI and cetuximab versus FOLFOX and panitumumab. Therefore, in clinical practice, the choice of cetuximab versus panitumumab with FOLFIRI or FOLFOX is made based on clinical goal (i.e., aiming towards liver resection), patient factors such as age and co-morbidity, and QOL factors (e.g, hair loss), in addition to the risk of allergic reaction to the mAb. Additionally, another phase III study evaluated the role of panitumumab in conjunction with FOLFIRI as a second-line treatment for mCRC [70]. This trial consisted of 1100 patients who had previously undergone one fluoropyrimidine-based treatment regimen. Preliminary safety data indicated that 15% of patients experienced neutropenia and 9% had diarrhoea. Furthermore, 12% reported grade 3 skin reactions, while grade 4 skin toxicities were almost negligible, seen in less than 1% of participants. Treatment of CRC, including the targeted therapies, is routinely updated through ASCO guidelines [71].

##### Other Anti-EGFR mAbs

There are several other anti-EGFR mAbs that are currently in preclinical and clinical developmental stages in various solid tumours, including CRC. These have been summarised in Table 1.

##### EGFR TKIs

Small-molecule TKIs targeting the ATP-binding site of the EGFR tyrosine kinase domain are promising agents for cancer treatment. They inhibit tyrosine phosphorylation via reversible or irreversible mechanisms, showing effectiveness in various cancers like lung and pancreatic [25,28] (Figure 1). They have been extensively studied in CRC.

##### Gefitinib (Iressa)

The first orally active selective EGF inhibitor was approved by the FDA for advanced non-small cell lung cancer (NSCLC) [25] (Table 1). Preclinical studies showed it inhibited colorectal tumour cell growth and reduced EGFR, pEGFR, pMAPK, and pAKT levels [72]. However, clinical trials in CRC yielded limited success [73,74,75,76,77,78,79,80,81]. Early studies with gefitinib and oxaliplatin showed inactivity [82], and a phase II trial found it inactive as a single agent [83]. While combinations with 5-FU, leucovorin, and oxaliplatin showed some promise [75,76], gefitinib is not currently in CRC trials.

##### Erlotinib (Tarceva™)

Erlotinib is another reversible EGFR inhibitor, FDA-approved for NSCLC and pancreatic cancer, and is in advanced development for multiple other cancer types, including colorectal [28,84]. Unlike gefitinib, erlotinib has displayed some efficacy as a monotherapy in CRC [85]. Various studies have explored its role alongside standard chemotherapy, with mixed results. In particular, a phase II study combining erlotinib with capecitabine and oxaliplatin showed promising partial responses in previously treated patients [86]. Another study pairing erlotinib with FOLFOX and bevacizumab faced challenges due to toxicity [41]. A phase I trial reported a high response rate when erlotinib was combined with FOLFOX4 and bevacizumab, albeit with notable side effects [87]. Interestingly, the dual EGFR targeting strategy combining erlotinib with cetuximab indicated enhanced inhibition of the EGFR pathway, achieving a 31% response rate, particularly in KRAS wild-type patients, with fewer adverse effects than expected [88]. More recently, clinical trials further investigating erlotinib in combination therapies with anti-EGFR monoclonal antibodies and other agents, found varying degrees of success. Erlotinib in combination with bevacizumab and FOLFOX4 compared to bevacizumab and FOLFOX4 alone yielded better clinical outcomes (i.e., longer PFS and better partial response and stable disease rate) in patients with mCRC; however, adverse effects were high, as with previous phase I studies [89]. Interestingly, in one study, a combination of bevacizumab and erlotinib was found to have favourable activity in patients with CRC, independent of RAS status [90]. On the other hand, a more recent study investigating the effects of erlotinib treatment in CRC cell lines found that there is significantly more downregulation of autophagy proteins in cells that were KRAS-mutated (HCT116) compared to those with wild-type KRAS (HKe3) [91].

#### 2.2.2. HER2 and HER3 Targeted Therapy

Overexpression of HER2 and HER3 has been reported in a variety of solid tumours, including CRC, and as such, these receptors are ideal targets for therapy with monoclonal antibodies, small-molecule TKIs in combination with chemotherapeutic interventions. However, advancements in the development of anti-HER2 or HER3 has been slower than expected. Indeed, the irreversible HER2 TKI tucatinib is the only HER TKI that has been approved in combination with anti-HER2 mAb trastuzumab for the treatment of patients with advanced unresectable or metastatic chemo-refractory HER2-positive RAS wild-type mCRC (please see later) [52,92,93]. There are several others that are in various stages of preclinical and clinical development, some of which will be discussed here and summarised in Table 1.

##### Trastuzumab (Herceptin)

Trastuzumab, a humanised mAb against the extracellular domain of the HER2 receptor, was the first HER antibody approved for the treatment of patients with HER2-overexpressing breast (2004) and, subsequently, stomach cancers [94]. In CRC, early clinical studies suggested a relatively lower expression of HER2 at 8%, hence a less attractive target for therapy with anti-HER2 inhibitors [95]. However, a number of preclinical studies using a panel of human colorectal tumour cell lines found trastuzumab as a single agent to be effective in reducing HER2 protein levels in colon cancer cell lines [96]. In other studies, trastuzumab was found to markedly increase radiographic response in mCRC patients harbouring HER2 gene amplification [47].

HER2 Amplification for Colorectal Cancer Enhanced Stratification (HERACLES) was an important phase II study as it proved the concept that blockade of HER2 can be achieved by trastuzumab and lapatinib (a dual EGFR and HER2 TKI) in HER2-positive mCRC. The objective response rate of 30% and disease control rate of 70% was impressive, as this was a heavily pre-treated group of patients with chemo-refractory disease [97]. This study led the way to others, including HERACLES-B [98] and later DESTINY-CRC01 [99], which investigated the efficacy of pertuzumab, an anti-HER2 mAb, and trastuzumab-emantasine (T-DM1, an antibody drug conjugate of trastuzumab and cytotoxic re-agent emtansine, a derivative of maytansine) and found objective response rates of 45.3%, with acceptable side effects. Currently, several clinical trials involving pertuzumab and CRC are at various stages of completion (NCT05786716, NCT03225937, NCT06136897, NCT03365882) (Table 1). However, more recently, trastuzumab, when used in combination with HER-2 TKI tucatinib, has gained FDA approval for the treatment of CRC.

##### Tucatinib

Tucatinib, a selective HER2 TKI, has recently emerged as a promising agent in combination with trastuzumab for patients with mCRC. Tucatinib binds to the TKI domain of HER2, blocking the signalling pathways and consequently preventing tumour proliferation. While earlier studies using HER2-positive CRC patient-derived xenograft models demonstrated significant tumour suppression, more recent findings notably showed single-agent tucatinib to have limited efficacy [100]. The pivotal phase II MOUNTAINEER study confirmed an objective response rate (ORR) of approximately 32%, with durable responses observed in patients with chemotherapy-refractory, HER2-positive, RAS wild-type mCRC receiving the combination of tucatinib and trastuzumab, leading to their approval by the FDA in 2023 [101]. More recently, the phase III MOUNTAINEER-03 study investigated the efficacy of tucatinib, trastuzumab, and mFOLFOX6 as a first-line treatment in HER2-positive mCRC. Preliminary findings suggest that this combination could further improve outcomes in treatment-naïve patients [102].

These studies have provided exciting developments in the armamentarium of treatment options for clinicians treating mCRC. However, it is important to recognise that only 2–5% of cases of mCRC show overexpression or amplification of HER2. It is, therefore, only a small percentage of mCRC patients who will potentially benefit from these anti-HER2 agents, but this makes it even more vital to identify these patients through molecular profiling in order for them to reap the benefits of these targeted agents.

Unlike therapies against EGFR and HER2 in CRC, which have approved regimens, anti-HER3 treatments are still very much in the early stages of development. This is partly due to the very weak tyrosine kinase activity of HER3, which led to it previously not being considered an important therapeutic target compared to EGFR and HER2. However, several studies have since demonstrated a key role of HER3 in the activation of the PI3K/AKT signalling pathway in EGFR, HER2, and the hepatocyte growth factor receptor, MET, addicted tumours, and in particular, resistance to HER inhibitors such as cetuximab [103,104,105,106,107]. In the following section, the results of investigation with various types of HER3 inhibitors will be briefly highlighted.

##### Seribantumab (MM-121)

Seribantumab (MM-121) is a fully humanised mAb and the first selective HER3 antagonist, inhibiting the activation of HER3 and downstream signalling pathways implicated in tumour growth and survival (Table 1). Some studies suggested that combination therapy with other agents (e.g., EGFR inhibitors) may improve response rates in HER3-expressing CRC patients [108]. In earlier studies, MM-121 was found to effectively inhibit the ligand-dependent activation of HER3 induced by either EGFR, HER2, or MET, and subsequently leading to restored sensitivity to anti-EGFR therapies by preventing reactivation of ErbB3 [109]. However, more recent clinical trials have yielded mixed results in solid tumours, including CRC. A phase I study combining the HER3 antibody and cetuximab with or without irinotecan found that, while the doublet combination of seribantumab and cetuximab was well tolerated, only 1/34 patients previously untreated with EGFR therapy had a partial response [110]. Another phase I dose escalation study of seribantumab monotherapy in patients with advanced solid tumours, majority CRC, found that the best response was stable disease in 24% and 39% of patients during dose escalation and expansion portions of the study, respectively [111]. These results suggest that there is therapeutic potential for seribantumab in some cancers and further studies can be justified to validate its role in CRC treatment.

##### Patritumab (U3-1287)

Patritumab (U3-1287) is a fully human mAb that targets HER3 by blocking the binding of neuregulin (NRG) on the receptor, leading to inhibition of the proliferation and survival of tumour cells [112]. Patritumab was evaluated in two early phase I clinical trials assessing its safety, pharmacokinetics, and early efficacy in advanced solid tumours (NCT00730470 and NCT01957280). The Western trial (NCT00730470) included 29 patients with metastatic CRC, but no objective responses (complete or partial responses) were reported, indicating limited efficacy [113]. Similarly, in the Asian trial (NCT01957280), only two patients with CRC were enrolled, and neither showed any signs of tumour regression [112]. Despite the acceptable safety profile, the lack of clinical responses in CRC led to the discontinuation of further development of patritumab in this indication.

To enhance therapeutic potential, an ADC version, patritumab deruxtecan (U3-1402), was developed. This agent combines patritumab with a topoisomerase I inhibitor (DX-8951 derivative) to improve cytotoxic efficacy [114]. Although preclinical studies showed promising results, a phase II clinical trial (NCT04479436) in advanced or metastatic CRC was terminated early after failing to meet pre-specified efficacy criteria. While patritumab itself as a monotherapy did not advance in CRC treatment, its development provided critical insights into HER3 biology and therapeutic resistance mechanisms, shaping the direction for next-generation HER3-targeted strategies. Indeed, there are several recent studies investigating the efficacy of patritumab deruxtecan (HER3-Dxd) in various solid tumours such as HER2-negative breast cancer and EGFR-mutated NSCLC [115,116,117,118,119,120,121,122,123,124,125,126].

##### GSK2849330

GSK2849330 is an anti-HER3 mAb designed to block HER3 signalling and potentially overcome resistance to EGFR-targeted therapies. While there are ongoing efforts to explore HER3 inhibition in CRC, the direct evaluation of GSK2849330 in CRC patients remains sparse. Studies indicate that dual HER2/HER3 targeting might be a promising therapeutic avenue [127]. In December of 2024, the FDA granted accelerated approval of the first anti-HER2/HER3 bispecific IgG1 antibody, zenocutuzumab (Bizengri), for the treatment of patients with pancreatic adenocarcinoma and NSCLC harbouring *NRG1* gene mutation [128,129]. Further studies should investigate the therapeutic application of this antibody by its repurposing in the treatment of CRC patients with co-expression of HER2/HER3.

##### Lumretuzumab

Lumretuzumab, another anti-HER3 mAb, has been investigated in combination therapies to enhance anti-tumour activity. Research suggests that anti-HER3 therapies could play a role in overcoming resistance to standard EGFR inhibitors in CRC [106,130].

#### 2.2.3. Dual- and Pan-HER Inhibitors of EGFR Family Members

The development of resistance to HER-targeted monotherapy is indeed a large challenge to overcome. This, along with the relatively small population of patients whose tumours are found to be sensitive to treatment with a single HER inhibitor, has stimulated research on the development of a number of dual- and pan-HER inhibitors, some of the main ones are discussed here, and others summarised in Table 1.

##### Lapatinib

Lapatinib, a dual tyrosine kinase inhibitor targeting EGFR and HER2, has drawn attention for its potential role in CRC treatment. While primarily approved for HER2-positive breast cancer, research into its application in CRC, particularly in metastatic and HER2-positive cases, has expanded in recent years. Several studies have reported the therapeutic potential of lapatinib as a single agent and in combination with other chemotherapeutic agents, such as that discussed above, the blockade of HER2 achieved by trastuzumab and lapatinib in the HERACLES study [97]. A 2025 study by Sun et al. explored lapatinib’s role in inducing ferroptosis and apoptosis in colon cancer cells, highlighting a potential new mechanism of action for this drug in CRC treatment [131]. Lapatinib’s role in CRC treatment remains under investigation in clinical trials, with the goal of defining patient subgroups that may benefit most from this therapy. Efforts are ongoing to identify predictive biomarkers, improve combination strategies, and assess long-term safety and efficacy [92] (Table 1).

##### Afatinib

Afatinib is an example of an irreversible TKI targeting pan-HER (EGFR, HER2, and HER4) approved for NSCLC with EGFR mutations [48,132]. Early experimental models found afatinib to be an effective and selective irreversible inhibitor of EGFR and HER2/neu [133].

We previously assessed the effects of afatinib, both as a monotherapy and in combination with standard cytotoxic agents or the anti-EGFR monoclonal antibody ICR62, on a panel of human colorectal cancer cell lines. Afatinib markedly inhibited the growth of EGFR-overexpressing DiFi cells (IC_50_ = 45 nM) and showed activity across other colorectal tumour lines, with IC_50_ values ranging from 0.33 µM in CCL-221 cells to 1.62 µM in HCT-116 cells. Of note, co-expression of EGFR, HER2, and HER3 correlated significantly with sensitivity to afatinib (*p* = 0.021) [46]. However, in a randomised, open-label phase II trial comparing afatinib with cetuximab in patients with mCRC, afatinib demonstrated inferior efficacy to cetuximab in KRAS wild-type tumours, and only modest disease control in KRAS-mutated tumours [134]. Similar findings of limited clinical efficacy were noted in a more recent phase I study of afatinib plus the MEK inhibitor selumetinib in patients with KRAS-mutant, PIK3CA wild-type colorectal tumours [135].

Currently, afatinib is under preclinical and clinical investigation for the treatment of several cancers, including CRC [136] (NCT03785249).

##### Neratinib

Neratinib is another irreversible pan-HER inhibitor targeting EGFR, HER2, and HER4, currently approved for the treatment of HER2-positive breast cancer and currently under clinical development for other tumours, including CRC [137]. A recent study investigated the combination of neratinib with cetuximab in quadruple-WT (KRAS, NRAS, BRAF, PIK3CA) metastatic CRC patients who had previously shown resistance to cetuximab or panitumumab. The findings suggest that the neratinib-plus-cetuximab regimen may enhance sensitivity in this subgroup, indicating potential for targeted therapy [138]. Several preclinical and clinical trials are evaluating neratinib across multiple tumour types, including CRC [139,140].

### 2.3. Development of Other Targeted Therapies Against CRC

In addition to the above-mentioned anti-HER family inhibitors, there has been significant ongoing development in targeting CRC with other classes of drugs, some of which have been approved by the FDA for the treatment of patients with CRC, as summarised in Table 2 below.

Of note, the recent advances in the treatment of BRAF V600E-mutant mCRC have established encorafenib and cetuximab as a foundational targeted therapy, particularly following the pivotal BEACON CRC trial [141]. The latest phase III BREAKWATER trial has extended these findings, investigating the combination of encorafenib and cetuximab with or without mFOLFOX6 as a first-line therapy. Preliminary results demonstrated that this combination, with or without chemotherapy, is effective and well-tolerated in untreated BRAF V600E-mutant mCRC patients, marking a potential shift toward chemotherapy-sparing regimens in selected cases [142]. In addition, evidence from another phase III trial confirmed the efficacy of encorafenib plus cetuximab in the first-line setting and reinforced the clinical utility of dual inhibition targeting BRAF and EGFR pathways [143]. Together, these studies refine the positioning of encorafenib and cetuximab in the treatment regimen for mCRC and highlight the evolving role of targeted regimens in both pre-treated and treatment-naïve patients. The growing consensus suggests that encorafenib plus cetuximab, with or without chemotherapy, offers a personalised, mutation-guided strategy that is reshaping clinical standards of care in BRAF-mutant mCRC.

Similarly, the emergence of KRAS G12C inhibitors has reshaped the therapeutic landscape for, albeit, a small subset of increasingly clinically relevant CRC patients. Among these, sotorasib in combination with panitumumab has shown promising clinical activity in KRAS G12C-mutated metastatic CRC and recently gained FDA approval (Table 2). The CodeBreaK 101 phase 1b study demonstrated that this combination yielded an ORR of 30% and a disease control rate (DCR) of 93% in heavily pre-treated patients with manageable toxicity, suggesting an actionable strategy for a population historically considered undruggable [144].

Follow-up data from the CodeBreaK 300 phase III trial compared sotorasib plus panitumumab against standard care in chemorefractory KRAS G12C-mutant CRC, with early findings supporting improved progression-free survival and safety [145]. Notably, sotorasib monotherapy has shown limited efficacy in CRC compared to lung cancer, presumably due to adaptive feedback via EGFR signalling. Hence, dual blockade using panitumumab is essential for sustained MAPK pathway suppression, as reinforced by multiple trials [146]. This evolving evidence base supports the integration of sotorasib and panitumumab in both second-line and potentially first-line treatment regimens for KRAS G12C-mutant CRC patients.

#### New Generation of Targeted Therapies Currently in Clinical Development

It is evident that despite all the current advancements in the development of targeted therapies, there are several challenges, as discussed below in Section 3. Therefore, recent clinical research highlights a growing number of trials exploring a newer generation of targeted therapies, such as bispecific antibodies, ADCs, and CAR-T cell therapies in CRC to circumvent the issues faced with current therapies, discussed briefly below and summarised in Table 3.

For bispecific antibodies, agents like SSGJ-707, ivonescimab (anti-PD-1/VEGF, NCT06959550), MCLA-129 (EGFR/c-MET, NCT04868877, and NCT04930432), and B1962 (anti-PD-L1/VEGF, NCT06724263) are under active investigation globally. Phase III entries include ivonescimab and AK129 (PD-1/LAG-3, NCT06943820), reflecting progression in clinical validation. In the ADC category, investigational therapies include M9140-targeting CEACAM5 (NCT06806046, NCT05464030), Dato-Dxd (Trop-2-targeting, NCT05489211), and MGC026 (NCT06242470). These are being tested both as monotherapies and in combination with checkpoint inhibitors (e.g., disitamabvedotin with tislelizumab in NCT05350917). CAR-T cell therapies in CRC are also rapidly expanding. Notable candidates include Claudin18.2 CAR-T (NCT06946615), CEA-targeted CAR-Ts (e.g., NCT05415475, NCT06821048), and novel logic-gated or allogeneic CAR-T platforms such as A2B694 (NCT06051695) and A2B395 (NCT06682793). LGR5-targeted CAR-T (NCT05759728) and HER2-targeted CAR-T combined with oncolytic viruses (NCT03740256) reflect cutting-edge strategies (Table 3).

## 3. Challenges in Targeting CRC

### 3.1. Patient Selection for a Better Response to Anti-EGFR Therapy

A major factor impacting the response rate to the targeted therapies is the availability of biomarkers for the identification of a more specific subpopulation of patients who can benefit from such therapeutic interventions. Patient selection for the targeted therapy of CRC has been an ongoing challenge since the approval of the first anti-EGFR mAbs, cetuximab and, subsequently, panitumumab.

Indeed, although these mAbs were approved for the treatment of CRC patients expressing EGFR, no significant correlation was found between the expression of EGFR and clinical response [27,147]. As a result, focus shifted towards evaluating factors downstream of the extracellular receptor domain, which revealed KRAS mutations to be a major contributor to resistance to EGFR-targeted therapies [148,149].

In 2009, the FDA approved amended labels of both cetuximab and panitumumab for use in patients whose tumours excluded KRAS mutation in exon 2 codon 12 and 13. As a result, in August 2009, NICE (UK) approved the use of cetuximab in combination with either FOLFOX or FOLFIRI for patients presenting with liver-only metastases from KRAS wild-type tumours, where the metastases were not amenable to surgical resection and could not be sufficiently downstaged with initial chemotherapy. However, despite excluding for this KRAS mutation, a significant number of eligible patients failed to respond to the anti-EGFR treatments, and further investigations found that other rare mutations in KRAS, BRAF, NRAS, and PIK3CA were also associated with lower response rates to anti-EGFR treatments in these patients [150,151]. The presence of the BRAF V600E mutation has important clinical significance, as it heavily dictates treatment strategies. This mutation occurs in about 10–15% of mCRC cases and is associated with a subgroup of patients who have a poor response to standard first-line chemotherapy and a poor prognosis [152,153]. It is well recognised to be a strong negative predictor of response to panitumumab and cetuximab, the two main anti-EGFR inhibitors in common clinical practice. This was demonstrated by the meta-analysis by Pietrantonio et al. [154]. The presence of the BRAF V600E mutation in mCRC generally precludes the use of an anti-EGRF mAb with the conventional doublet chemotherapy, such as FOLFOX or FOLFIRI, due to the resistance to these agents and the low RR. The BEACON trial was a phase III study looking at the outcomes of patients with BRAF mutations previously treated with chemotherapy when treated with cetuximab and a BRAF inhibitor, encorafenib, compared to those patients receiving standard chemotherapy (the control arm) [141,142,143]. The results confirmed a significant improvement in median OS in the group of patients receiving encorafenib and cetuximab (8.4 months) compared to the control group (5.4 months). The safety profile was favourable, and this doublet has now become the standard of care for patients in this setting.

More recently, additional negative predictive biomarkers of response to anti-EGFR treatments in mCRC patients, such as HER2 and MET amplifications, PTEN loss, and right-sided primary CRC, have been identified and summarised in the latest guidelines of the National Comprehensive Cancer Network (NCCN) [155,156,157,158,159,160]. However, it is noteworthy that while these mutations can predict a negative response to therapy with anti-EGFR mAbs in some patients, objective responses of up to 44% have been reported in studies investigating mCRC patients with KRAS mutations treated with cetuximab, and only a 41% response rate is observed in patients with left-sided tumours [156,161].

### 3.2. Variable Expression of the HER Family in CRC

#### 3.2.1. Expression of EGFR

As discussed above, despite the approval of mAbs cetuximab and panitumumab for the treatment of patients with mCRC, there was no significant association between the immunohistochemical expression of EGFR and response to therapy in various studies. Indeed, there is a wide variation in the expression of EGFR in the literature, which ranges between 8% and 100% [32,162,163] (Table 4).

There are several factors that could have contributed to this variability, and as a result, the unexpected lack of relationship between expression of EGFR and limited response to anti-EGFR therapies. A deeper analysis of the trials leading to the approval of the anti-EGFR mAbs cetuximab and panitumumab shows that these studies predominantly used the PharmDx™ kit to determine the expression of EGFR [27], which has been shown not to differentiate between wtEGFR or a mutated form of EGFR [164]. In addition, in one of the largest investigations, Jolien and colleagues analysed EGFR status in 755 patients with mCRC using the PharmDx assay. Applying a low threshold for positivity as membranous staining in more than 1% of tumour cells, they reported EGFR expression in 61.7% of cases. However, this biomarker status showed no predictive value for cetuximab efficacy [165]. In contrast, another study investigating the association between the expression of wtEGFR using a specific anti-wtEGFR antibody and response to cetuximab in mCRC patients found that cytoplasmic expression of wtEGFR was associated with a poor response to cetuximab therapy [166] (Table 4).

This highlights the impact of some of the contributing factors, such as choice of antibody and staining criteria, e.g., cut-off thresholds to determine positive staining, leading to variation in the immunohistochemical expression of EGFR and consequently rendering it an unreliable predictive biomarker for response to treatment with anti-EGFR targeted therapies. Notwithstanding this, it is important to note that tumours are indeed heterogenous in nature and both inter- and intra-tumour heterogeneity could also contribute to the variability in the reported expression of EGFR and the lack of association with response to EGFR therapies, as well as discordance between the expression of EGFR in the primary tumour and its metastatic lesions [167]. A previous study investigating 144 RAS wild-type mCRC patients found that of the 21 patients with primary and paired metastasis, in 38% of cases examined both the primary tumour and related metastasis were EGFR-negative (staining present in less than 5% of tumour cells or staining intensity of 0) and 48% (10/21) of patients had discordance in the expression of wtEGFR in the primary tumours and related metastasis [93].

Despite the variability in the percentage of EGFR-positive cases across various studies, EGFR protein continues to be an optimal target for therapeutic intervention with anti-EGFR antibodies. IHC remains the predominant method for assessing EGFR expression levels and their cellular localisation. Nevertheless, the absence of a standardised scoring system, variations in antibody usage, differences in patient demographics, and, as previously highlighted, the small sample size in certain studies contribute to the observed variability in EGFR expression patterns and the proportion of EGFR-positive cases in CRC patients, as summarised in Table 4.

**Table 4:** Studies investigating the expression pattern of EGF receptor protein and its ligands in CRC.

Study	Number of Patients	Tumour Type	Percentage Expression (%)
[168]	92	Dukes’ A–D	16.3
[169]	32	Dukes’ A–D	44
[170]	82	Dukes’ A–D	97.6
[171]	102	Stage IV	75.5
[172]	249	Dukes’ C and D	72.7
[173]	125	Dukes’ A–D	53
[174]	99	Primary/met	53
[162]	244	Stage 0–IV	8
[175]	80	Stage IV	80
[176]	150	Primary/met	97
[177]	158	Primary/met	85 (primary) 79 (met)
[178]	87	Dukes’ C	76
[179]	32	Stage IV	84
[180]	154	Dukes’ A–D	35.6
[32]	27	Metastatic	100
[181]	130	Stage I–IIB	73
[182]	106	Primary/met	12.3/54.7
[183]	124	Stage I–IV	60
[184]	164	Stage I–IV	43.9
[185]	109	Stage IIA–IIIC	57.8
[165]	755	Metastatic	61.7
[186]	101	Metastatic	89
[187]	205	Stage III	80.5
[188]	147	Stage I–IV	35.7
[189]	54	Stage I–IV	58
[163]	86	Dukes’ A–D	43
[190]	158	Grades G1–G4	38
[191]	70	Stage I–IV	64
[192]	173	Stage III	62.4
[193]	331	Stage I–IV	39

Consequently, to address these inconsistencies, it is crucial to undertake more comprehensive studies examining the expression levels of EGFR ligands, various isoforms of EGFR, and other members of the HER family, along with their cellular localisation in a larger cohort of patients, utilising a standardised approach to scoring criteria.

#### 3.2.2. Expression of HER2

Extensive research has documented aberrant HER2 expression across multiple tumour types, most notably in breast cancer. Anti-HER2 therapies are already approved for use in patients with HER2-overexpressing early-stage and metastatic breast cancer, as well as in metastatic gastric cancer and several other malignancies, as outlined earlier. In colorectal cancer, HER2 expression has been widely examined using methods such as IHC and FISH, with key findings summarised in Table 5 below.

It is noteworthy that, like the expression pattern of EGFR discussed above, there is a wide variation in the expression level of HER2 reported in patients with CRC. Several earlier studies, such as those by Osako et al. [194], Kruszewski et al. [195], and Kavanagh et al. [196], reported HER2 expression rates ranging from 1.8% to 70% in CRC patients.

**Table 5:** Studies investigating the expression levels of HER2 in CRC.

Study	No. of Patients	Tumour Type	Method of Assessment	HER2 Expression (%)
[173]	125	Dukes’ A–D	IHC	35
[197]	169	Stage I–IV	IHC	3.6
			FISH	2.4
[198]	170	Dukes’ B–C	IHC	87 (cyto)
				54 (mem)
[162]	244	Stage 0–IV	IHC	3
[95]	138	Metastatic	IHC	8
[199]	87	Dukes’ C	IHC	89
[200]	77	Dukes’ A–D	IHC	30
[201]	137	Stage I–IV	IHC	47.4
			FISH	1.45
[183]	124	Stage I–IV	IHC	27.4
[196]	132	Dukes’ A–D	IHC	13.6
[202]	186	Dukes’ A–D	FISH	26.3
[165]	755	Metastatic	FISH	11.5
[195]	202	Primary	IHC	66 (cyto) 27 (mem)
[203]	60	Primary node positive	IHC	1.8
[204]	365 174	Stage I–IV	IHC	6
			IHC	5.8
			SISH	6.3
[205]	208	Stage I–IV	IHC	8.2
[93]	144	Metastatic	IHC	97

However, more recent investigations, including work by Khelwatty et al. [93,163,166], have provided updated insights into HER2’s clinical significance. Our 2021 [93] study examined 144 patients with metastatic CRC and found that HER2 expression correlated with a shorter PFS in patients treated with anti-EGFR monoclonal antibodies such as cetuximab and panitumumab. Specifically, HER2 expression was detected in 97% of cases, with membranous HER2 being linked to worse PFS, while cytoplasmic HER2 expression was associated with better outcomes. Additionally, discordance in HER2 expression between primary and metastatic tumours was observed in 71% of cases, highlighting the complexity of HER2 as a biomarker in mCRC treatment [93].

#### 3.2.3. Expression of HER3 and HER4

HER3 (ErbB3) is a unique member of the HER family due to its weak intrinsic kinase activity. It relies on dimerisation with other HER receptors, such as HER2 or EGFR, to drive oncogenic signalling. The presence of HER3 is particularly notable in left-sided CRC, where it is linked to aggressive tumour features, resistance to anti-EGFR therapies, and poor prognosis [206]. This is because HER3 can activate the PI3K/AKT and MAPK signalling pathways, both of which contribute to cell survival and proliferation. Additionally, HER3 expression increases in response to anti-EGFR treatment, providing a potential escape mechanism for tumours that develop resistance, discussed in more detail in Section 3.3 below. Despite its strong role in tumour progression, the expression of HER3 reported in CRC ranges from 16 to 89%, making it a more frequently observed marker than HER2 but also exhibiting the same wide variation in expression [103,163,173,203,207,208,209] (Table 6). However, unlike EGFR and HER2, HER3 remains a challenging therapeutic target; hence, with the exception of the recently approved bispecific antibody targeting both HER2 and HER3 discussed above (see Section 2.2.2), there is no clinically approved HER3-specific mAb for the treatment of cancer patients [128,129,210].

HER4 (ErbB4) is the least understood member of the HER family in CRC. Unlike HER2 and HER3, which are often implicated in tumour progression, HER4 has a more complex role, sometimes acting as an oncogene and other times as a tumour suppressor, depending on the cellular context and isoform expression. Studies on HER4 in CRC have been limited, but some evidence suggests that its expression is associated with tumour differentiation and favourable prognosis [211]. HER4 can undergo proteolytic cleavage, releasing an intracellular domain that translocates to the nucleus and influences gene transcription. This mechanism is thought to contribute to cellular differentiation rather than proliferation. In contrast, some reports indicate that HER4 expression can promote CRC progression under certain conditions, especially in tumours co-expressing HER2 or EGFR. The presence of HER4 may facilitate compensatory signalling in response to targeted therapies, contributing to resistance mechanisms [212].

Despite these observations, HER4 remains underexplored in CRC research, with very few studies investigating the expression of HER4 in CRC, ranging from 11 to 92% of the cases examined [163,173,185,211,213] (Table 6). More studies are needed to clarify its role in tumour biology and therapeutic resistance. Additionally, identifying specific HER4 isoforms that may drive oncogenic signalling versus those that contribute to tumour suppression could be key to its clinical relevance.

**Table 6:** Studies investigating the expression levels of HER3 and HER4 and co-expression all HER family members in CRC.

Study	No. of Patients	Tumour Type	Markers Assessed	Percentage Expression (%)
[209]	55	Dukes’ A–C	HER3	78
[173]	125	Dukes’ A–D	EGFR	52
			HER2	35
			HER3	36
			HER4	22
[211]	106	Dukes’ B–D	HER3	17 (mem), 28.3 (cyto)
			HER4	18.9 (mem), 30.2 (cyto)
[213]	64	Stage I–III	EGFR	76
			HER2	54
			HER3	67
			HER4	81
[185]	109	Stage IIA–IIIC	EGFR	57.8
			HER2	8.3
			HER3	69.7
			HER4	11 (mem), 19.3 (cyto)
[203]	60	Primary/met	EGFR	29
			HER2	1.8
			HER3	16
[163]	86	Primary	EGFR	43
			HER2	77
			HER3	52
			HER4	92
[205]	208	Liver met	HER3	75
[193]	331	Stage I–IV	HER3	69
[93]	144	Metastatic	EGFR	25
			HER2	97
			HER3	79
			HER4	48

#### 3.2.4. Co-Expression of All HER Family Members

Indeed, very few studies have investigated the role of all four HER family members (EGFR/HER1, HER2, HER3, and HER4) in CRC, shedding light on their prognostic and therapeutic significance [163,173,185,213] (Table 6). Indeed, it is vital to study the co-expression of all HER family members, as tumours are heterogenous and there exists an immense degree of cross-talk between these receptors. Ye et al. [212] reviewed targeted therapies for CRC, emphasising the potential of HER3 inhibition, particularly through drugs like patritumab deruxtecan (U3-1402) as promising therapeutic options. Similarly, Desai et al. [214] explored HER-family-targeted therapies, focusing on strategies to block EGFR, HER2, and HER4 dimerization, suggesting their relevance in developing new treatment approaches.

Furthermore, we have also highlighted the prognostic significance of HER family expression in CRC, reporting that co-expression of EGFR and HER4 correlates with poorer disease-free survival in advanced-stage CRC patients, indicating their potential as prognostic biomarkers [163]. In contrast, Kountourakis et al. [211] found that HER3 and HER4 were frequently overexpressed in CRC, with HER4 expression being linked to better prognosis, suggesting a complex role in tumour progression. Meanwhile, Cao et al. [215] further investigated expression of HER3 and HER4, concluding that HER3, but not HER4, was significantly associated with clinicopathological features and patient prognosis, reinforcing HER3’s role as a critical prognostic marker.

These results and the discussions throughout this review underscore the intricate roles of HER family members in CRC. While EGFR and HER2 remain established therapeutic targets, HER3 has emerged as a significant prognostic marker and potential drug target. HER4, despite its association with both tumour progression and improved survival in some contexts, warrants further investigation to clarify its dual role. The growing interest in HER3-targeted therapies and the resistance mechanisms associated with HER family inhibitors highlight the need for continued research and development of combination therapeutic approaches in CRC treatment.

On the mechanistic front, Jia et al. [216] identified HER4 as a promoter of CRC progression through the induction of epithelial/mesenchymal transition (EMT), underscoring its potential as a therapeutic target. Similarly, Ahcene Djaballah et al. [217] reviewed the development of HER2-targeted therapies, reaffirming HER2 amplification as a viable treatment target in CRC. However, therapeutic resistance through adaptive resistance pathways remains a challenge, supporting the necessity of combination treatment strategies to overcome resistance [218]. In 2002, Lee et al. analysed the expression of all HER family members in 125 Dukes’ A–D CRC cases. While overexpression of any single HER receptor was not significantly linked to reduced overall survival, concurrent overexpression of HER2 and HER4 correlated with a poorer prognosis [173]. Similarly, Baiocchi et al. evaluated 109 stage II–III CRC patients for EGFR through HER4 expression. Their findings revealed that membranous HER4 positivity was an independent predictor of recurrence, whereas the absence of HER3 expression independently predicted both higher recurrence risk and worse survival in multivariate analysis [185].

### 3.3. Acquired Drug Resistance to Therapies in CRC

Acquired drug resistance to targeted therapies in CRC remains a major challenge yet to be overcome. Indeed, as discussed above, there are several genetic aberrations, such as mutation in KRAS, NRAS, BRAF, and PIK3CA, loss of PTEN, and amplification of HER2/MET, which have been associated with the de novo resistance to anti-EGFR therapies in CRC. However, in most cases, these “passenger biomarkers” are extremely rare and comprise a very small percentage of the CRC patient population [219,220].

In addition to these genetic aberrations, there are several non-genetic mechanisms that have been shown to contribute to acquired resistance to EGFR therapies. For example, some early in vitro studies have shown the overexpression of EGFR ligands to play a role in the survival of tumour cells against anti-EGFR therapies [221,222].

More recently, we showed the impact of EGFR ligands on response to treatment with cetuximab in a clinical study of 60 patients with mCRC. We found a significant association between the expression of EGFR ligands BTC, epigen, and worse overall survival. In addition, amphiregulin expression was significantly associated with poorer progression-free survival, while the co-expression of wild-type EGFR with any one of its ligands was significantly associated with shorter progression-free survival [166]. There is mounting evidence supporting EGFR ligands, which are overexpressed in CRC, with potential key roles in tumour progression, as predictive biomarkers for EGFR-targeted therapy sensitivity, as well as mediators of therapy resistance, reviewed in more detail by Guernsey–Biddle and colleagues [223]. Similarly, in another study, the HER3 ligand, heregulin, was found at elevated levels in patients with stable or progressive disease compared to those with a response to cetuximab and, hence, thought to drive the resistance against cetuximab [224]. Indeed, using in vitro analysis of EGFR overexpressing CRC parental (DiFi) and drug-resistant variant (DiFi62) cells, we have previously shown enhanced activation of both HER2 and HER3 as a novel mechanism of drug resistance against anti-EGFR mAbs ICR62 and cetuximab and highlighted the therapeutic benefits of using pan-HER inhibitors following the development of acquired resistance to EGFR antibody-based therapy [106].

Further investigation of the relative expression and cellular location of all members of the HER family and their ligands in a larger group of CRC are warranted and should help to improve the response rate and development of resistance to HER inhibitors by using drugs targeting one or more member of the HER family in combination with other approved targeted agents by repurposing them [225].

## 4. Future Directions and Concluding Remarks

In summary, this review comprehensively examines the advancements in targeted therapies for CRC, focusing primarily on HER family members and their inhibitors. Significant progress has been achieved following the development and approval of the first generation of anti-HER agents, including mAbs like cetuximab and panitumumab, and necitumumab. These agents have demonstrably improved treatment outcomes for specific patient sub-groups, particularly those with wild-type KRAS status, expanding the therapeutic arsenal against this challenging malignancy. Clinical trials incorporating these agents and the newer generation of HER inhibitors in combination with other targeted therapeutics and conventional chemotherapies have further led to improved survival rates in patients with CRC, and in particular, patients with primary or acquired resistance to therapy, as summarised in the sections above. However, the clinical application of targeted therapies in CRC remains hampered by several key challenges.

The most significant obstacle is the identification of reliable biomarkers for patient selection. While initial optimism centred on EGFR expression as a predictive biomarker, subsequent research revealed that KRAS mutations are strongly associated with resistance to anti-EGFR therapies [148]. The FDA’s amended labels for cetuximab and panitumumab, restricting their use to KRAS wild-type patients, underscore this crucial limitation. Furthermore, other mutations (in NRAS, BRAF, PIK3CA, PTEN amplifications, HER2/MET, and right-sided primary CRC) have also been associated with reduced response rates, demonstrating the complexity of CRC’s underlying biology and the need for a more nuanced approach to patient stratification [150,151]. The high degree of intratumoral and intertumoral heterogeneity further complicates biomarker identification and the development of truly predictive tests. Variations in EGFR expression levels reported across different studies highlight the challenges in establishing standardised assessment methods and scoring criteria [166].

This heterogeneity underscores the limitations of current biomarker-based selection strategies and emphasises the need for multi-biomarker panels capable of more accurately identifying patients most likely to benefit from specific targeted therapies.

Overcoming these challenges requires a multifaceted approach. Future research should focus on discovering new and more robust biomarkers that can better predict treatment responses, potentially using liquid biopsies and preclinical models such as patient-derived xenografts for real-time tumour progression and response [226]. Advanced genomic and proteomic analyses could identify novel molecular targets that could be exploited for therapeutic intervention. Furthermore, research should prioritise the development of novel therapeutic agents, including next-generation inhibitors targeting intracellular domains of HER family members or pan-HER inhibitors, which have shown promise in overcoming resistance mechanisms [227,228,229]. Combination therapies, combining multiple targeted agents or combining targeted therapies with immunotherapy, as well as drug repurposing and biosimilars, hold significant promise in enhancing treatment efficacy and circumventing drug resistance [225,230,231,232,233,234]. In addition, a rapidly evolving area in CRC therapy involves the use of nanocarrier-based drug delivery systems. Liposomes, polymeric nanoparticles, micelles, and ADC have shown promise in improving drug solubility, prolonging systemic circulation, and enhancing tumour-specific uptake [235,236]. These delivery vehicles can increase the therapeutic index of conventional and targeted agents by reducing off-target toxicity and enabling controlled drug release within the tumour microenvironment. Emerging strategies also explore ligand- or antibody-functionalised nanoparticles to selectively target HER2-positive or EGFR-overexpressing CRC cells [237]. Incorporating nanotechnology into future clinical trial designs may further enhance the safety and efficacy of targeted therapies in CRC.

The development of companion diagnostic tests that accurately stratify patients based on multiple biomarkers is essential for maximising the clinical utility of targeted therapies. Such testing will enable more precise patient selection and reduce the incidence of adverse events and associated toxicities with ineffective treatment. Furthermore, the development of less toxic therapies that can be administered in more convenient ways may encourage more widespread adoption of targeted approaches.

In conclusion, the field of CRC treatment is evolving rapidly, driven by technological advancements and increasing knowledge of the disease’s intricate biology. Targeted therapies represent a significant advance over conventional treatments, but challenges remain. A multifaceted approach focusing on refined patient selection strategies, innovative therapeutic agents, and comprehensive biomarker testing will be crucial to fully realising the promise of these approaches and improving treatment outcomes for patients with CRC. Ongoing and future clinical trials employing combination therapies, novel inhibitors, and improved diagnostic approaches are key to unlocking the full therapeutic potential of targeted therapies by improving treatment outcomes in patients with mCRC.

## Figures and Tables

**Table 2:** Summary table of other FDA-approved drugs for the treatment of patients with CRC.

Drug/Combination	Target	FDA Approval Date	Reference
Bevacizumab (Avastin)	VEGF-A antibody	2004	Standard use; included in NCI list
Ziv-aflibercept (Zaltrap)	VEGF-trap fusion	3 August 2012	VELOUR Phase III
Regorafenib (Stivarga)	Multi-kinase TKI	27 September 2012	CORRECT Phase III
Ramucirumab (Cyramza)	VEGFR-2 antibody	24 April 2015	RAISE trial Phase III
Pembrolizumab (Keytruda)	PD-1 inhibitor	June 2020 (adult)	KEYNOTE-177 (1st-line) standard
Fruquintinib (Fruzaqla)	VEGFR1-3 TKI	8 November 2023	FRESCO-2 Phase III
Adagrasib (Krazati)	KRAS G12C inhibitor	January 2024 (colorectal)	KRYSTAL-1 (NCT03785249)
Encorafenib (Braftovi) + Cetuximab ± binimetinib	BRAF V600E + EGFR ± MEK	1 October 2024 (with FOLFOX)	BEACON-CRC Phase III
Sotorasib (Lumakras) + Panitumumab	KRAS G12C + EGFR	Jan 16, 2025	CodeBreaK-300 Phase III
Nivolumab (Opdivo) ± Ipilimumab (Yervoy)	PD-1 ± CTLA-4 inhibitors	2 May 2025 (combo); Monotherapy since 2017	CheckMate-8HW Phase III

**Table 3:** Summary of the other class of drugs currently in clinical trials for the treatment of patients with CRC.

NCT Number	Drug/Title	Status	Target(s)
**Bispecific Antibodies**			
NCT06493760	SSGJ-707	Recruiting	PD-1/VEGF
NCT06959550	Ivonescimab (PD-1/VEGF)	Not yet recruiting	PD-1/VEGF
NCT06838546	B1962 Injection	Not yet recruiting	PDL-1/VEGF
NCT04868877	MCLA-129	Recruiting	EGFR/c-MET
NCT06663839	NILK-2301	Recruiting	CEACAM5/CD3
NCT05985109	KN 046 + Regorafenib	Recruiting	PD-L1/CTLA-4
NCT04606472	SI-B003	Recruiting	PD-1/CTLA-4
NCT03526835	MCLA-158	Recruiting	EGFR/LGR5
NCT05780307	IMM2520	Recruiting	PD-L1/CD47
NCT04603287	SI-B001	Recruiting	EGFR/HER3
NCT04930432	MCLA-129	Recruiting	EGFR/c-MET
NCT05985707	KN026 ± KN-046 + Chemo	Not yet recruiting	HER2
NCT06621563	HS-20117 combo	Recruiting	EGFR/cMET
NCT06943820	AK129 combo	Recruiting	PD-1/LAG-3
NCT05411133	Cabotamig (ARB202)	Recruiting	CDH17/CD3
NCT07044908	TQB2922 + TAS-102 ± Bev	Not yet recruiting	EGFR/cMET
NCT06724263	B1962 (anti-PD-L1/VEGF)	Not yet recruiting	PD-L1/VEGF
NCT06790212	Ivonescimab + CAPOX	Not yet recruiting	PD-1/VEGF
NCT05426005	Cadonilimab	Recruiting	PD-1/PD-L1
NCT06147037	[225Ac]-FPI-2068	Recruiting	EGFR/cMET
**Antibody/Drug Conjugates (ADCs)**			
NCT06806046	M9140 (Anti-CEACAM5)	Recruiting	CEACAM5
NCT06825624	HS-20093 combinations	Recruiting	B7-H3 (CD276)
NCT05350917	Tislelizumab + DisitamabVedotin + Pyrotinib	Not yet recruiting	HER2
NCT05464030	M9140 (Anti-CEACAM5)	Recruiting	CEACAM5
NCT06242470	MGC026	Recruiting	B7-H3
NCT06781983	IPH4502	Recruiting	Nectin-4
NCT05489211	Dato-Dxd (TROPION-PanTumor03)	Recruiting	Trop-2
**CAR-T cell Therapies**			
NCT06946615	Claudin18.2 CAR-DC/CAR-T	Recruiting	Claudin18.2
NCT06718738	IM96 CAR-T	Recruiting	GUCY2C
NCT05415475	CEA-CAR-T	Recruiting	CEA
NCT06055439	CHM-2101 (Cadherin 17)	Recruiting	CDH17
NCT06653010	Universal CAR-T (REVO-UWD-01)	Recruiting	GUCY2C
NCT06821048	CEA-CAR-T	Recruiting	CEA
NCT05240950	CEA-CAR-T for Liver Metastases	Recruiting	CEA
NCT06043466	CEA-CAR-T	Recruiting	CEA
NCT06675513	WD-01 Autologous CAR-T	Recruiting	GUCY2C
NCT06885697	Anti-Mesothelin CAR-T (TNhYP218)	Recruiting	Mesothelin
NCT05028933	IMC001	Recruiting	EpCAM
NCT06051695	A2B694 (Logic-gated CAR-T)	Recruiting	MSLN, HLA-A*02-
NCT06682793	A2B395 (Allogeneic, EGFR)	Recruiting	EGFR, HLA-A*02-
NCT03740256	Oncolytic Virus + HER2-CAR-T	Recruiting	HER2
NCT06937567	CDH17 CAR-T	Recruiting	CDH17
NCT06256055	UCMYM802 (Mesothelin+)	Recruiting	Mesothelin
NCT05759728	CNA3103 (LGR5-CAR-T)	Recruiting	LGR5
NCT05639972	E7 TCR-T	Recruiting	HPV E7
NCT06885424	A2 Bio Gene Therapy Follow-up	Not yet recruiting	CEA/MSLN
NCT03692429	alloSHRINK (CYAD-101)	Recruiting	NKG2D
NCT04991948	CYAD-101 + Pembrolizumab + FOLFOX	Recruiting	NKG2D
NCT05736731	A2B530	Active, not recruiting *	CEA/-HLA-A*02

* granted FDA orphaned drug designation.
